# Postoperative lung herniation following minimally invasive septal myectomy: A rare case report with successful surgical repair

**DOI:** 10.1016/j.ijscr.2025.111313

**Published:** 2025-04-17

**Authors:** Mohammed Saleh, Islam Jadallah, Qais Alhroub, Maaweya Jabareen, Wasef Alhroub, Ammar Hassouneh

**Affiliations:** Faculty of Medicine, Hebron University, Hebron, Palestine

**Keywords:** Lung herniation, Minimally invasive septal myectomy, Hypertrophic obstructive cardiomyopathy, Post-surgical complications

## Abstract

**Background:**

Lung herniation is a rare condition where lung tissue protrudes through the thoracic wall, often due to surgery, trauma, or pressure changes. The intercostal type is most common, especially after minimally invasive cardiac surgery (MICS) like septal myectomy for HOCM. Early diagnosis and treatment are crucial to prevent complications like respiratory distress and lung strangulation.

**Case presentation:**

A 57-year-old male with cardiac disease underwent minimally invasive septal myectomy for HOCM. Ten days later, he developed shortness of breath, dry cough, and a right chest mass. Imaging studies, including a chest X-ray revealed a triangular lucency lateral to the right 4th and 5th ribs and non-contrast CT confirmed lung herniation due to an intercostal muscle tear. He underwent surgical repair with rib plating and mesh, leading to significant improvement. He was discharged after seven days and remained asymptomatic at 8-month follow-up.

**Discussion:**

Lung herniation after MICS is rare but serious, caused by intercostal muscle injury. Risk factors include lung conditions, pressure changes, and surgery. Small cases may resolve, but larger ones need surgery. Timely imaging and rib plating with mesh prevented complications and ensured recovery.

**Conclusion:**

Lung herniation should be considered in post-MICS patients with respiratory distress and chest abnormalities. Early diagnosis and surgery are crucial to prevent complications. This case emphasizes the importance of prompt recognition and proper surgical management.

## Highlights

• Lung herniation is an uncommon but serious complication following minimally invasive cardiac surgery (MICS), often resulting from intercostal muscle injury.
• Lung herniation presents with respiratory distress, a bulging chest mass, and diminished breath sounds. Chest X-ray and CT are essential for diagnosis.
• Symptomatic lung herniation requires surgical intervention.
• Heavy Smoking, lung disease, and high intrathoracic pressure increase lung herniation risk.

## 1 Introduction

Lung herniation is an uncommon clinical condition characterized by the protrusion of lung tissue and pleural membranes through an abnormal defect in the thoracic wall [1]. It was first described by Roland in 1499 and later classified by Morel-Lavallée into congenital and acquired types based on its cause and cervical, intercostal, and diaphragmatic types based on its location. Acquired cases are further categorized into traumatic, post-surgical, and spontaneous herniations [2].

The intercostal subtype, where the lung protrudes through intercostal muscle abnormalities, accounts for 60–80 % of instances and is frequently associated with trauma, surgery, or increased intrathoracic pressure owing to coughing or straining. Risk factors include central obesity, male gender, smoking, COPD, and steroid use [3]. While smaller, asymptomatic cases may heal on their own, larger or symptomatic herniations can cause chest pain, shortness of breath, or noticeable bulging at the hernia site, necessitating surgical correction by defect closure, mesh placement, or rib stabilization [4].

Minimally invasive cardiac surgery (MICS), which occurred in the mid-1990s for mitral valve repair, has since been widely used for treating mitral and tricuspid valve disease as well as performing operations like surgical septal myectomy for hypertrophic obstructive cardiomyopathy [5]. This approach, frequently performed via mini-sternotomy or right lateral thoracotomy, offers substantial advantages over complete sternotomy, including fewer wound healing issues and faster recovery. However, MICS is not without risks; lung herniation has been identified as a rare but significant complication [6].

We report the case of a 57-year-old male with a history of diabetes and hyperlipidemia who presented with chest pain and shortness of breath. He was diagnosed with lung herniation following a septal myectomy performed for hypertrophic obstructive cardiomyopathy. A CT scan confirmed lung herniation caused by an intercostal muscle tear. The defect was surgically repaired, and the patient was discharged after seven days with improved respiratory function.

This work has been reported in line with the SCARE criteria [7].

## 2 Case presentation

A 57-year-old Palestinian male with a history of heavy smoking, diabetes, cardiac disease, and hyperlipidemia presented with worsening exertional chest pain and shortness of breath. Despite treatment with nitrates, his symptoms persisted. His family history was notable for coronary artery disease (CAD). Physical examination revealed a forceful apical impulse, a bifid carotid pulse, a harsh systolic murmur best heard at the left lower sternal border, S4 heart sound, and right soft subcutaneous swelling that protrude with coughing. His BMI was 23.6 kg/m^2^**.**

Given the patient's symptoms of chest pain, shortness of breath, and respiratory distress, the differential diagnosis included pulmonary embolism, pneumothorax, diaphragmatic hernia, and traumatic lung injury. However, these conditions were ruled out based on the clinical history and physical examination.

ECG was done and showed evidence of left ventricular hypertrophy, with tall R waves in the left precordial leads (V5, V6) and deep S waves in the right precordial leads (V1, V2). T-wave inversions are observed in the lateral leads (V5, V6). Additionally, mild ST-segment depression is noted in the inferior leads (II, III, aVF).

An echocardiogram showed asymmetrical septal hypertrophy, left ventricular outflow tract obstruction, and hyperdynamic left ventricular function, consistent with hypertrophic obstructive cardiomyopathy (HOCM). Cardiac catheterization revealed a completely occluded obtuse marginal branch 1 (OM1) with left-to-left collateral circulation. The right coronary artery (RCA) was patent, with mild in-stent restenosis (ISR) noted in a previously placed stent extending from the ostium to the mid-RCA.

The patient subsequently underwent minimally invasive septal myectomy for HOCM. Ten days post-procedure, he returned with complaints of shortness of breath and a persistent dry cough. On examination, a right soft, bulging mass was observed on the chest wall, becoming more pronounced with inspiration, coughing, or straining. Palpation revealed a tender, reducible mass, while auscultation indicated diminished breath sounds over the affected area. The overlying skin was intact without signs of erythema or infection. Mild respiratory distress and subtle asymmetry in chest wall movement were also noted. Initial laboratory tests were within normal limits.

**Fig. 1:**
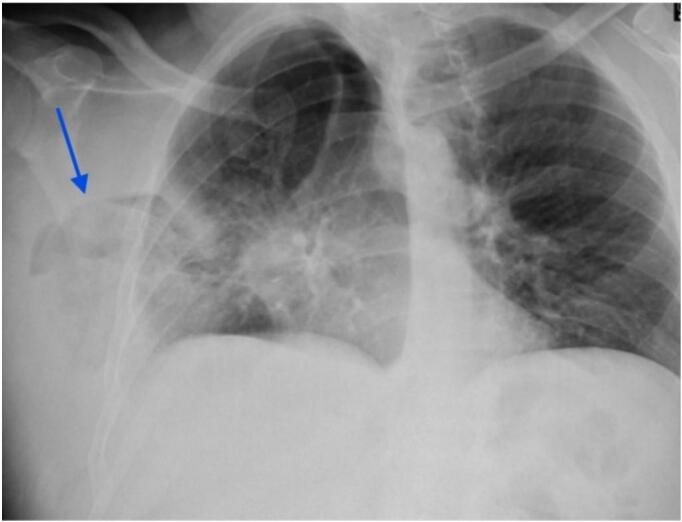
The Chest X-ray (PA view) showed a triangular lucency (blue arrow) lateral to the right 4th and 5th ribs, indicating lung protrusion beyond the thoracic cage. (For interpretation of the references to colour in this figure legend, the reader is referred to the web version of this article.)

**Fig. 2:**
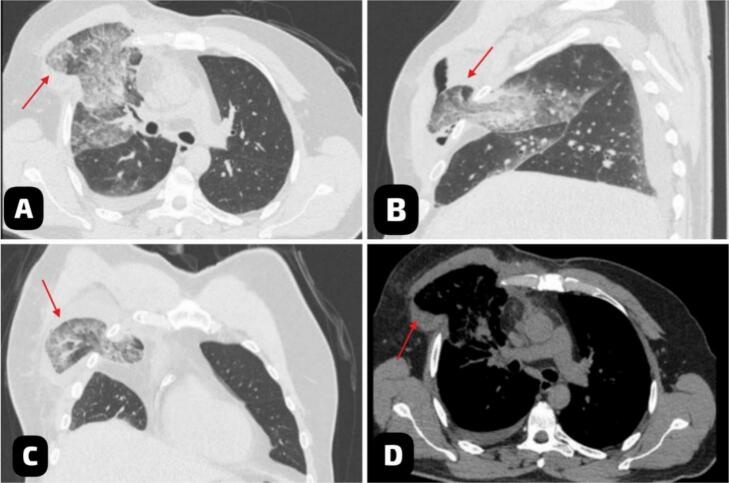
Multiplanar chest CT scan without IV contrast in lung windows (A, B, C) and mediastinal window (D) showed intercostal herniation of right lung parenchyma (red arrow). (For interpretation of the references to colour in this figure legend, the reader is referred to the web version of this article.)

Vital signs at presentation showed a heart rate of 92 bpm, blood pressure of 124/72 mmHg, and oxygen saturation of 88 % on room air. The patient exhibited cyanosis, tachypnea, and reduced breath sounds over the left lower chest, along with a systolic murmur on auscultation. A chest X-ray revealed a triangular lucency lateral to the right 4th and 5th ribs (Fig. 1). This finding was further clarified by a non-contrast chest CT, which showed herniation of the right lower lung lobe due to an intercostal muscle tear, likely iatrogenic. The imaging raised concerns about potential lung strangulation or infarction (Fig. 2).

The patient was promptly taken to surgery, where rib plating and mesh reinforcement were performed to close the defect and stabilize the thoracic wall. Prophylactic vancomycin powder was applied intraoperatively to minimize the risk of infection**,** especially after the addition of the mesh. Postoperatively, the patient's respiratory status improved significantly, with oxygen saturation (SpO2) increasing to 95 % on room air. He was closely monitored in the ICU for 48 h and discharged on postoperative day 7 with instructions for wound care and pulmonary rehabilitation.

At an 8-month follow-up, the patient remained asymptomatic, with normal vital signs and no evidence of recurrence or complications.

## 3 Discussion

Hernial protrusions of the lung are exceptionally rare, with limited literature available on the subject. Notably, pulmonary hernia was first documented by Dr. Roland in 1499 [8,9]. While trauma has the primary association with lung herniation, it can also be considered an uncommon postoperative complication related to minimally invasive cardiac or thoracic surgery. The incidence of lung herniation after cardiac surgery has remained low, but it becomes an important consideration because of potential significant morbidity if left untreated [2,3]. Symptomatic hernias may present with dyspnea, chest wall pain, or a visible or palpable chest bulge, which is most commonly observed in intercostal lung hernias. However, they can also be asymptomatic [10].

Lung herniation is diagnosed using imaging techniques, primarily chest radiographs and computed tomography (CT), which help visualize the herniated lung and identify potential consequences, such as lung tissue strangling and infarction [11,12].

Minimally invasive cardiac surgery has become very popular due to the benefits it offers, including shorter hospital stays, faster recovery times, and better cosmetic outcomes. However, the reduced invasiveness of MICS may also be a factor in the increased risk of thoracic wall instability, particularly in patients with predisposing conditions such as chronic obstructive pulmonary disease, extended steroid use, or prior thoracic trauma [4,12].

Few cases in the literature describe lung herniation following minimally invasive cardiac procedures. Wiedemann et al. reported a rare instance of lung herniation after minimally invasive atrial septal defect closure, attributed to weakened intercostal structures or increased intrathoracic pressure from the surgical technique. Diagnosis was achieved through imaging, and surgical repair was required to restore chest wall integrity [13]. Similarly, Gulielmos et al. described symptomatic lung herniation in a 71-year-old man after minimally invasive coronary artery bypass grafting. Symptoms included chest wall pain, respiratory discomfort, and a visible bulge exacerbated by coughing or straining. Diagnosis was confirmed by CT, which outlined the herniation and excluded complications such as lung strangulation [14].

In this case, the patient presented with chest wall pain, discomfort in breathing, and a bulge that was accentuated by coughing or straining following a minimally invasive cardiac procedure. Physical examination revealed a palpable defect in the chest wall, with imaging via CT confirming the diagnosis, demonstrating the extent of herniation.

The differential diagnosis for this patient includes pulmonary embolism, pneumothorax, diaphragmatic hernia, and traumatic lung injury, all of which could present with similar clinical features.

Management of lung herniation depends on symptom severity and the size of the defect. Asymptomatic cases can be treated conservatively, whereas symptomatic or large hernias usually need surgical treatment [2,6,11].

Intercostal hernias are often treated surgically, even if they are asymptomatic, to avoid consequences such as necrosis or defect extension. Conservative treatment may be attempted for supraclavicular hernias, as they frequently cure spontaneously, but surgery is suggested for more severe cases to assure better outcomes and prevent progression [2,5,6,11]. In our case, the surgical repair consisted of a combined closure of the defect with placing mesh in order to restore the integrity of the thoracic wall. The postoperative result was uneventful, with no perioperative complications or recurrences observed in long-term follow-up.

The rarity of lung herniation, added to its varied clinical presentations, makes timely diagnosis and early intervention very important. Preventive measures are important, especially emphasizing the need for meticulous closure techniques in surgical procedures and consideration of prophylactic measures, such as the use of biologic meshes and the use of prophylactic antibiotics, to decrease the risk of infection and recurrence. More studies and case reviews are needed to better understand effective treatment modalities and improve patient outcomes.

## 4 Conclusion

Lung herniation after minimally invasive myectomy is a rare yet serious complication that demands immediate recognition and management. Early diagnosis through imaging, particularly chest CT, played a key role in detecting the herniation and preventing severe complications like lung strangulation or infarction. Surgical repair, including rib plating and mesh reinforcement, effectively addressed the defect and stabilized the thoracic wall, resulting in marked improvement in the patient's respiratory function. This case underscores the importance of vigilant monitoring and swift intervention to manage postoperative complications effectively.

## CRediT authorship contribution statement

Mohammed Saleh handled conceptualization, data curation, and the writing of the original draft. Qais Alhroub handled writing and review. Islam Jadallah contributed to editing, and software. Maaweya Jabareen contributed to the investigation and visualization. Wasef Alhroub managed resources and validation. Dr. Ammar Hassouneh provided supervision.

## Informed consent

The patient provided written informed consent for the publication of this case report and accompanying images. A copy of the consent form is available for review by the Editor-in-Chief upon request.

## Ethical approval

This research did not require ethical approval, as the IRB committee does not mandate approval for reporting individual cases or case series.

## Guarantor

Qais Alhroub serves as the guarantor for this study, assuming full responsibility for the research and its outcomes. He had complete access to all the data and made the final decision to publish the study.

## Funding

This research was not supported by any specific grants from public, commercial, or not-for-profit funding agencies.

## Declaration of competing interest

None.

## Data Availability

All data supporting the study's findings are included in the article and are readily accessible.
